# Generative AI Models in Time-Varying Biomedical Data: Scoping Review

**DOI:** 10.2196/59792

**Published:** 2025-03-10

**Authors:** Rosemary He, Varuni Sarwal, Xinru Qiu, Yongwen Zhuang, Le Zhang, Yue Liu, Jeffrey Chiang

**Affiliations:** 1 Department of Computer Science University of California, Los Angeles Los Angeles, CA United States; 2 Department of Computational Medicine University of California, Los Angeles Los Angeles, CA United States; 3 Division of Biomedical Sciences School of Medicine University of California Riverside Riverside, CA United States; 4 Department of Biostatistics University of Michigan Ann Arbor, MI United States; 5 Institute for Integrative Genome Biology University of California Riverside Riverside, CA United States; 6 Institute for Cellular and Molecular Biology University of Texas at Austin Austin, TX United States; 7 Department of Neurosurgery David Geffen School of Medicine University of California, Los Angeles Los Angeles, CA United States

**Keywords:** generative artificial intelligence, artificial intelligence, time series, electronic health records, electronic medical records, systematic reviews, disease trajectory, machine learning, algorithms, forecasting

## Abstract

**Background:**

Trajectory modeling is a long-standing challenge in the application of computational methods to health care. In the age of big data, traditional statistical and machine learning methods do not achieve satisfactory results as they often fail to capture the complex underlying distributions of multimodal health data and long-term dependencies throughout medical histories. Recent advances in generative artificial intelligence (AI) have provided powerful tools to represent complex distributions and patterns with minimal underlying assumptions, with major impact in fields such as finance and environmental sciences, prompting researchers to apply these methods for disease modeling in health care.

**Objective:**

While AI methods have proven powerful, their application in clinical practice remains limited due to their highly complex nature. The proliferation of AI algorithms also poses a significant challenge for nondevelopers to track and incorporate these advances into clinical research and application. In this paper, we introduce basic concepts in generative AI and discuss current algorithms and how they can be applied to health care for practitioners with little background in computer science.

**Methods:**

We surveyed peer-reviewed papers on generative AI models with specific applications to time-series health data. Our search included single- and multimodal generative AI models that operated over structured and unstructured data, physiological waveforms, medical imaging, and multi-omics data. We introduce current generative AI methods, review their applications, and discuss their limitations and future directions in each data modality.

**Results:**

We followed the PRISMA-ScR (Preferred Reporting Items for Systematic Reviews and Meta-Analyses extension for Scoping Reviews) guidelines and reviewed 155 articles on generative AI applications to time-series health care data across modalities. Furthermore, we offer a systematic framework for clinicians to easily identify suitable AI methods for their data and task at hand.

**Conclusions:**

We reviewed and critiqued existing applications of generative AI to time-series health data with the aim of bridging the gap between computational methods and clinical application. We also identified the shortcomings of existing approaches and highlighted recent advances in generative AI that represent promising directions for health care modeling.

## Introduction

### Background

Generative artificial intelligence (GenAI) is a family of artificial intelligence (AI) models that can generate synthetic content ranging across text, images, audio, and video. Well-known GenAI tools such as ChatGPT [1] and DALL-E [1] have revolutionized the way in which people view AI [2]. On the other hand, data analysis in health care has entered a new era as large amounts of electronic health records (EHRs) are generated daily. Among these data, biomedical time series have been of particular interest to researchers in tasks including tracking patient trajectories [3] and treatment estimations [4]. Compared to cross-sectional data, which represent 1 time point, time series provide more information on development over time. There are 2 main types of time-series data: real-time and longitudinal data. While real-time data usually span a shorter period (minutes or days) and have more consistent time-point entries, longitudinal data are recorded over the magnitude of months or years with various sampling frequencies and number of entries. Compared to traditional time-series data in finance and climate science, biomedical time series face unique challenges in modeling, including missingness and sampling rate [5]. Therefore, traditional methods such as foundation models (FMs) that work well on high-frequency time series are not guaranteed to work well on biomedical data. In the age of big data, GenAI models have begun to outperform traditional statistical and machine learning (ML) methods in various tasks [6]. On the other hand, GenAI for health care faces unique challenges, including data privacy, transparency of the decision process, and other ethical issues that must be addressed. Nonetheless, these tools have the potential to improve disease modeling accuracy, tailor treatment plans across a heterogeneous population, and assist clinicians in making clinical decisions.

In this paper, we aim to provide a comprehensive review of GenAI applications to biomedical time-series data and a technical overview for clinicians who wish to work with AI tools but are unsure of where to begin. We survey GenAI applications to time-series health care data and its ability to improve patient care and the diagnosis process while considering the challenges and showcasing real-world uses that highlight its importance in the field. For the selection process, we followed the PRISMA-ScR (Preferred Reporting Items for Systematic Reviews and Meta-Analyses extension for Scoping Reviews; Multimedia Appendix 1) guidelines and searched in PubMed and Google Scholar for conference and workshop papers using the following keywords: “generative AI (GenAI),” “health care,” “time series,” “longitudinal,” “electronic health records (EHR),” “electronic medical records (EMR),” and “genetics.” From the initial search, we applied the following inclusion criteria—being peer reviewed, published after 2000, and focusing on time-series or longitudinal data—ultimately yielding 155 studies as of April 2024. The detailed selection process is described in the Related Works section.

**Figure 1:**
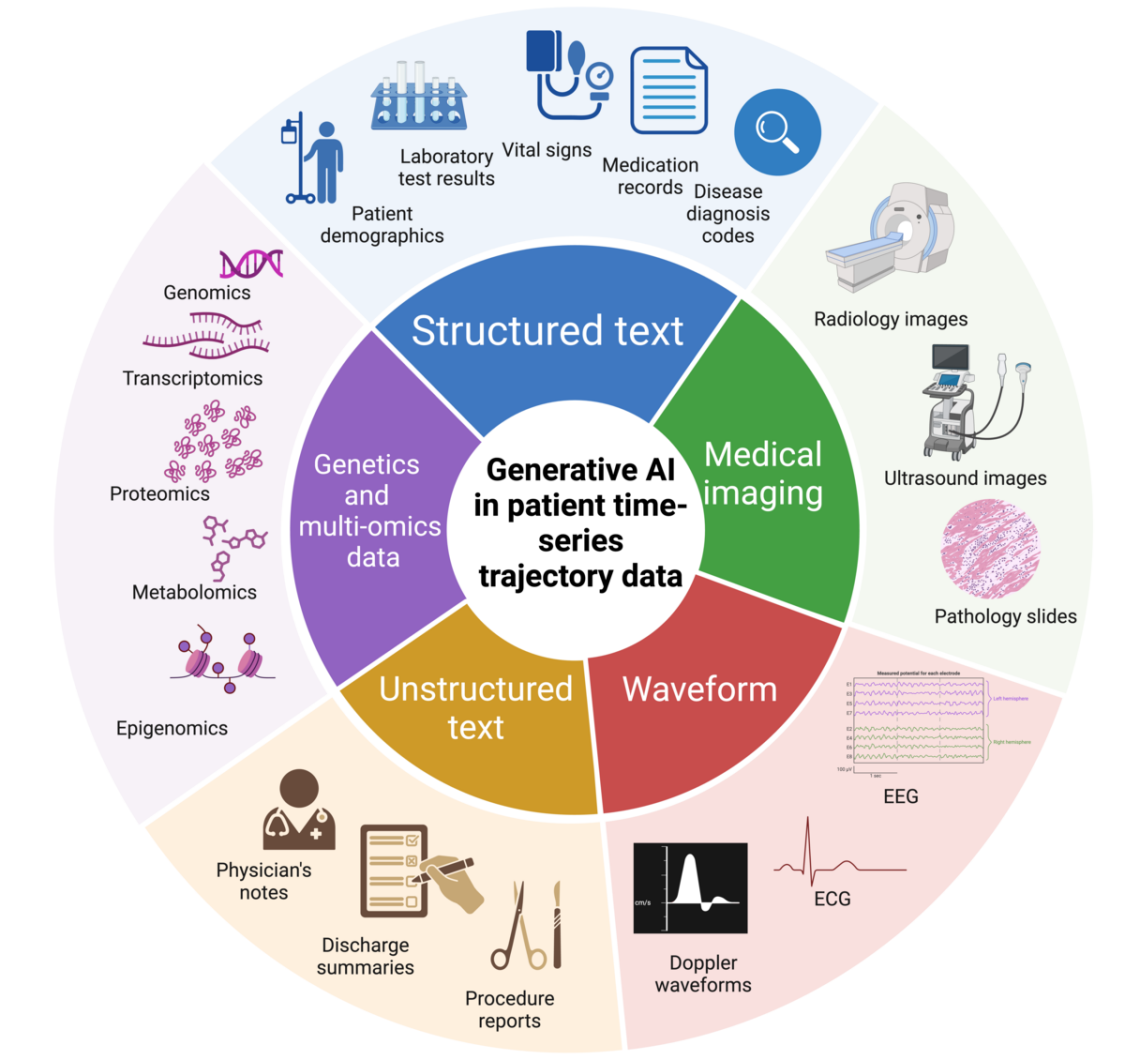
Health care data sources in different modalities for generative artificial intelligence (AI) application. ECG: electrocardiogram; EEG: electroencephalogram.

We organized the review by data modality, including structured and unstructured text, imaging, waveform, and multi-omics data, and illustrate possible data sources in each modality for GenAI application in Figure 1. For each modality, we discuss preprocessing, applications, model selection, and challenges and future directions. In the Model Selection sections, we help guide how to choose the appropriate models for this type of data for clinicians who are interested in incorporating GenAI into their practice. We organize the workflow for choosing an appropriate model for the data and computational resources at hand. We have organized the paper as follows: (1) overview of the algorithms and techniques introduced in the review; (2) structured data; (3) unstructured data; (4) medical imaging; (5) physiological waveforms; (6) genetics and multi-omics data; and (7) ethical considerations, challenges, and future directions.

### Related Works

**Figure 2:**
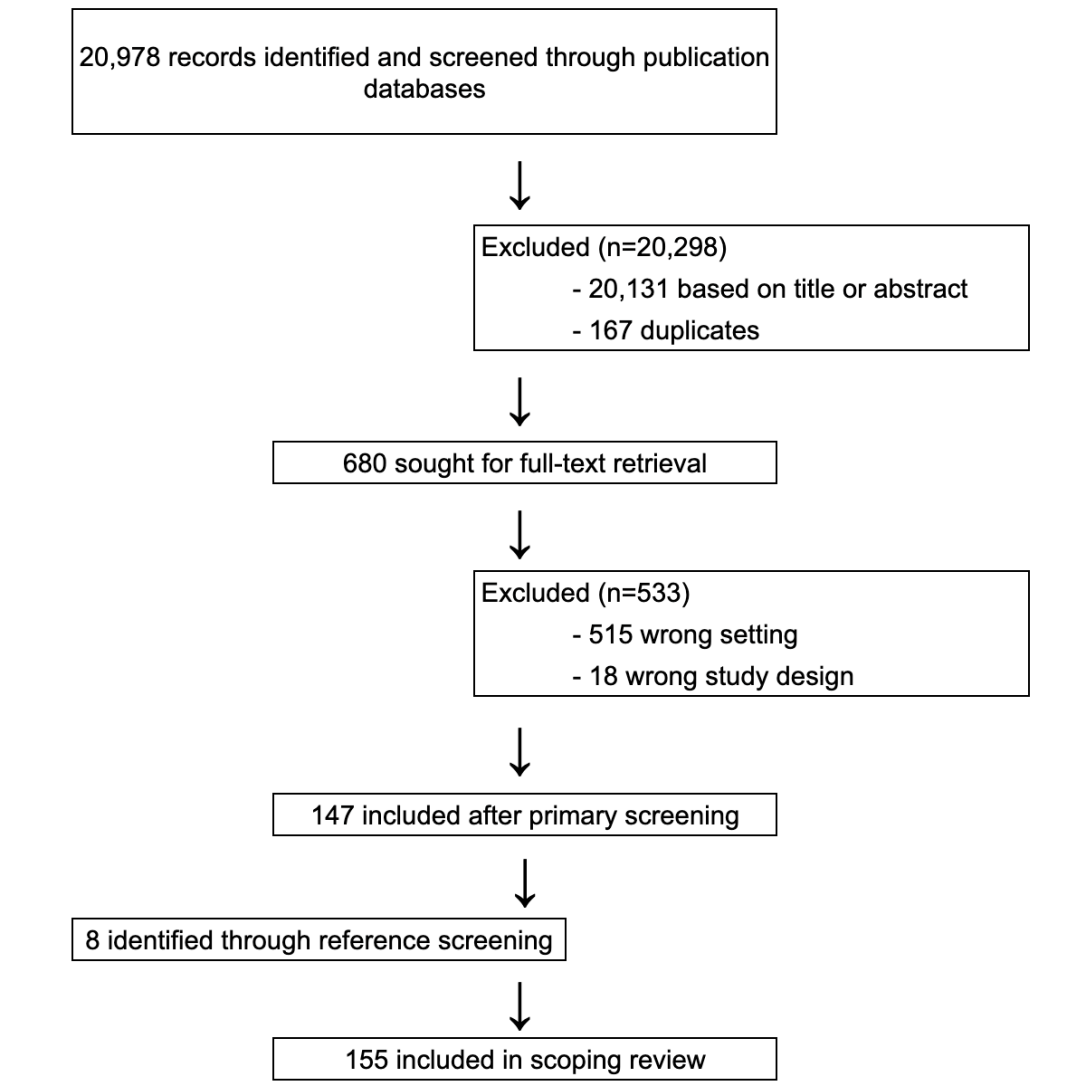
Study selection.

Our electronic search retrieved 20,978 study records: 20,003 (95.35%) from Google Scholar and 975 (4.65%) from PubMed, as shown in Figure 2. After title and abstract screening and removing duplicates, 3.24% (680/20,978) of these articles were retained for full-text review. Of these 680 articles, 533 (78.4%) were excluded, leaving 147 (21.6%) studies after the primary screening. A total of 8 more articles were added through secondary screening, resulting in a total of 155 articles included in our scoping review.

Our search for existing reviews on GenAI for disease trajectory modeling returned 39 entries broadly covering the use of AI in health care (Multimedia Appendix 2) in Google Scholar, Scopus, and PubMed. From a review of these works, although we observed an increasing number of AI-related review papers in the biomedical setting in recent years (Multimedia Appendix 3), there remained a gap in reviewing GenAI approaches working with time-series data specifically, prompting the need for our paper.

## Algorithm and Technique Overview

### Overview

**Figure 3:**
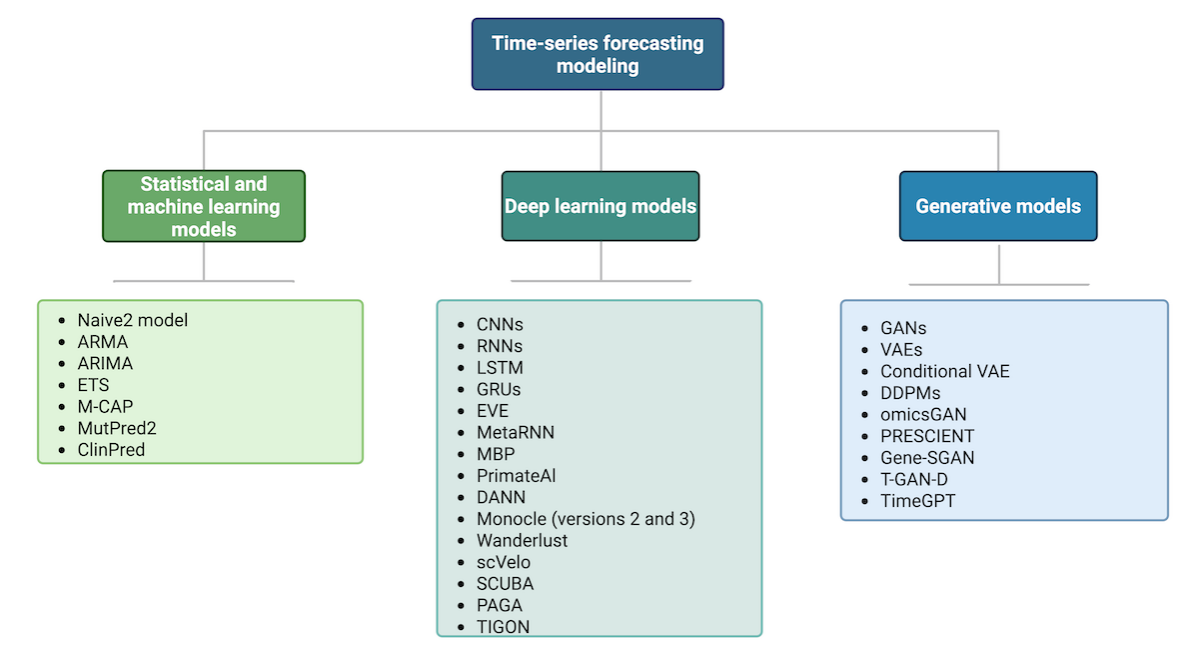
Existing models for time-series forecasting. ARIMA: autoregressive integrated moving average; ARMA: autoregressive moving average; CNN: convolutional neural network; DANN: deleterious annotation of genetic variants using neural networks; DDPM: denoising diffusion probabilistic model; ETS: exponential smoothing; EVE: Evolutionary Model of Variant Effect; GAN: generative adversarial network; GenAI: generative artificial intelligence; Gene-SGAN: gene-guided weakly supervised clustering via GANs; GRU: gated recurrent unit; LSTM: long short-term memory; M-CAP: Mendelian Clinically Applicable Pathogenicity; MBP: masked bidirectional prediction; PAGA: partition-based graph abstraction; PRESCIENT: Potential Energy Underlying Single-Cell Gradients; RNN: recurrent neural network; SCUBA: single-cell clustering using bifurcation analysis; T-GAN-D: Trained GAN Discriminator; TIGON: Trajectory Inference With Growth via Optimal Transport and Neural Network; VAE: variational autoencoder.

**Figure 4:**
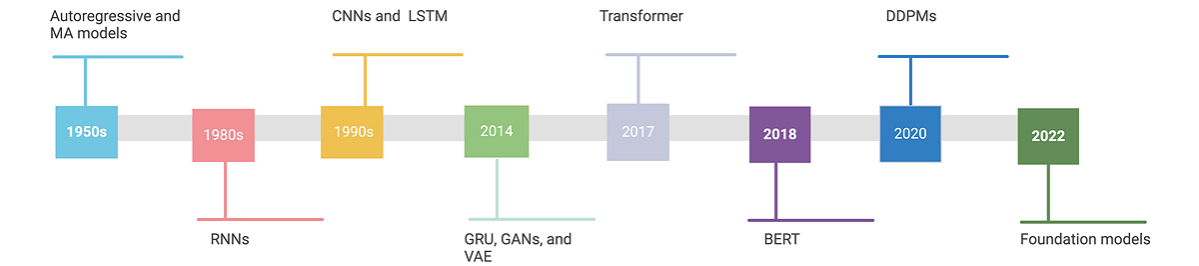
Milestones in time-series forecasting models. BERT: Bidirectional Encoder Representations From Transformers; CNN: convolutional neural network; DDPM: denoising diffusion probabilistic model; GAN: generative adversarial network; GRU: gated recurrent unit; LSTM: long short-term memory; MA: moving average; RNN: recurrent neural network; VAE: variational autoencoder.

Despite a shared nomenclature, it is important to differentiate between modern AI and the field of AI. The general study of AI encompasses the class of ML methods, which includes the subset of deep learning (DL) methods (also thought of as modern AI). Previous ML approaches have been mostly *discriminative* such that, given a set of input predictors, they learn a strategy (decision boundary) to fit or separate data points. On the other hand, DL models can be discriminative or generative based on the choice of the objective function. Therefore, they are not inherently generative or discriminative and are the building blocks of modern AI models, although they have typically been developed and demonstrated in the discriminative setting. Finally, GenAI methods belong to the class of *generative* ML methods and capture the underlying data generation process. By learning the distribution domain of the data, these models can synthesize hypothetical data points that are statistically indistinguishable from the originals. For discriminative tasks, GenAI models are often combined with downstream models, which can be simply rule based or more complex. In this section, we introduce a set of traditional, ML, DL, and GenAI models that are referenced later in the paper and list existing models used in time-series forecasting in Figure 3, as well as showing a timeline for when these models were first introduced in Figure 4 and a comparison among these methods using common metrics in Table 1.

**Table 1:** Metric comparison among methods for biomedical time-series prediction (1=lowest; 5=highest).

Model	Computational cost	Interpretability	Model size	Data requirement	Accuracy
Benchmark models	1	5	1	1	2
CNN^a^	2	3	2	3	3
RNN^b^	2	3	2	3	3
Transformer	3	4	3	3	5
GAN^c^	4	2	4	4	5
VAE^d^	2	3	3	4	4
Diffusion	2	1	3	4	5
NLP^e^	3	2	3	3	4
Foundation models	5	1	5	5	3

^a^CNN: convolutional neural network.

^b^RNN: recurrent neural network.

^c^GAN: generative adversarial network.

^d^VAE: variational autoencoder.

^e^NLP: natural language processing.

### Traditional Statistical Models

In this section, we introduce several statistical models commonly used as benchmarks in modeling time-series data. One of the most basic yet effective methods is the Naive2 model, which makes a prediction by using the value from the most recent observation or from a similar period in the past. In the class of autoregressive methods, the autoregressive moving average model [7] predicts the outcome value at a given time point using both a linear combination of multiple past observations and a linear combination of past error terms in the regression model. As an extension of the autoregressive moving average, the autoregressive integrated moving average (ARIMA) model [8] accounts for seasonal or periodical changes in the data. One of the most widely used methods is the Prophet model by Meta [9], which uses an additive model that accounts for yearly, weekly, and daily seasonality and holidays. Finally, the exponential smoothing (ETS) model [10] uses a weighted combination of multiple past observations in which the decay of the weights is exponentially distributed. While these methods have limited performance with highly complex data, they remain a popular choice due to their simplicity and transparency in the decision-making process.

### DL Models

DL models are the combination of an architecture (ie, the structure) and an objective (ie, the loss function). In this section, we introduce the canonical DL architectures that are the foundation of different GenAI settings but are not GenAI models themselves.

#### Convolutional Neural Networks

Convolutional neural networks (CNNs) [11] are a type of DL neural network architecture that is particularly well suited for processing and analyzing image data. CNNs are good at recognizing patterns in small areas and work well for short-term connections due to their ability to perform convolutions step by step over time [12]. CNNs can be used for univariate time-series forecasting, in which the trend can be viewed as a 2D graph. In addition, researchers have explored different CNN architectures for time-series forecasting, such as temporal convolutional networks [13] that are tailored for sequence-to-sequence modeling tasks, leveraging the strengths of CNNs while addressing the challenges of working with sequential data.

#### Recurrent Neural Networks

Recurrent neural networks (RNNs) [14] are one of the earliest DL frameworks that are designed to capture sequential dependencies and patterns across time steps. RNNs have an internal or hidden state that captures information from previous time steps and influences the processing of subsequent inputs. During training, sequential data are fed into the network one step at a time. The network processes each input along with its corresponding hidden state, updating the hidden state based on the current input and the previous hidden state. This process is repeated for each time step in the sequence, allowing the network to capture temporal dependencies and learn patterns in the data. The architecture of RNNs consists of multiple recurrent layers, each containing recurrent units. There are 2 main types of units: long short-term memory (LSTM) cells and gated recurrent units (GRUs). These units are responsible for capturing temporal dependencies and encoding information from previous time steps. Due to their training on sequential data and the leveraging of their recurrent structure, RNNs have performed strongly against traditional DL networks in modeling complex patterns in time-series data [15,16]. However, RNNs can be time-consuming as inputs are processed sequentially and do not model long-range dependencies well as the information diminishes over time.

#### Transformers

Transformers [17] are the foundation of many state-of-the-art generative models, including ChatGPT. Unlike RNNs, which process data sequentially and can be computationally expensive, transformers rely on a mechanism called self-attention [17] to capture dependencies simultaneously and over long ranges. They consist of an encoder-decoder architecture in which the encoder processes the input sequence and the decoder generates the output sequence. During training, the input is embedded into a high-dimensional space, and the encoder applies a self-attention mechanism to capture relationships between different parts of the input sequence in the embedding space. Self-attention allows each position in the sequence to attend to all other positions, enabling the model to learn contextual representations efficiently. These representations are then passed through feedforward neural networks within each layer to further process the information. The decoder, on the other hand, predicts the output sequence step by step based on the encoder’s contextualized representations and previous outputs. Transformer models typically include multiple layers of encoder and decoder blocks, each containing self-attention mechanisms and feedforward neural networks. In the age of large data, transformers are one of the most powerful models to capture complex relationships in sequential data. One example is in the field of natural language processing (NLP), where Bidirectional Encoder Representations From Transformers (BERT) [18] and generative pretrained transformer architecture have become state of the art, achieving the best performance across many NLP benchmarks [19,20].

### GenAI Models

#### Generative Adversarial Networks

A generative adversarial network (GAN) [21] is a generative vision model that consists of 2 parts: the generator that creates fake data and the discriminator that critiques them. The generator’s goal is to produce realistic outputs from random noise that resemble realistic images. On the other hand, the discriminator’s role is to differentiate between real data and the data generated by the generator. Through this back-and-forth process of creation and critique, GANs improve over time, producing increasingly realistic results. GANs are one of the earliest image generation models that have shown great success in generating synthetic images [22] and have been used extensively for the generation of new datasets and domain transfer in medical imaging [23,24]. However, training GANs can be challenging due to issues such as the limited type of data produced and instability during training.

#### Variational Autoencoders

Autoencoders are a class of neural network that learn a low-dimensional representation of high-dimensional structured data [25]. Autoencoders consist of 2 parts: an encoder that projects high-dimensional data into a latent space with lower dimensions and a decoder that learns to map a point in the latent space back to its high-dimensional representation. However, the latent distribution of a vanilla autoencoder is unknown, making inference difficult and prompting the need for variational autoencoders (VAEs). VAE models [26] make further assumptions about the sample generation process that allow us to model an approximation of the distribution of the latent space, usually a Gaussian distribution with a mean and variance estimated by the model. Unlike other image generation models, VAEs provide a latent space that can be estimated efficiently and used for further modeling. As with many medical applications, conditional variables offer additional information and can improve parameter estimation. A natural extension to the VAE is the conditional VAE, which includes additional conditional variables [27].

#### Diffusion Models

Denoising diffusion probabilistic models (DDPMs) [28] are a class of vision models that generate a new image by removing noise gradually from a pure noise input. The model learns to denoise data by understanding the probabilistic relationships between noisy and clean data points, making it particularly effective in scenarios in which data are corrupted by noise. During training, DDPMs simulate a diffusion process in which noise is gradually added to the data and then iteratively remove this noise to reconstruct the original signal. At each step, the noisy observation is generated by adding a small amount of noise to the previous observation. The diffusion process is typically modeled using an autoregressive process in which the next observation is conditioned on the previous observation and noise.

#### FMs for Medicine

FMs [29] are ML models capable of performing various generative tasks after being trained on extremely large and typically unlabeled datasets [30]. In the past few years, FMs have received significant attention given their impressive range of capabilities across multiple domains. FMs trained on EHRs have shown the ability to predict the risk of 30-day readmission [31], select future treatments [32], and diagnose rare diseases [33]. These models encode the patient features, such as procedures, diagnosis, medications, and laboratory values, into embeddings that represent the patient’s entire medical history. There are 2 broad categories of FMs built from electronic medical record data: clinical language models (CLaMs) and FMs for electronic medical records (FEMRs). CLaMs are a subtype of large language models that specialize on clinical or biomedical text—CLaMs are primarily trained on and output clinical or biomedical text. On the other hand, FEMRs are trained on the entire timeline of events in a patient’s medical history. Given a patient’s electronic medical record as input, an FEMR will output not clinical text but rather a machine-understandable representation for that patient. This representation, also referred to as a patient embedding, is typically a fixed-length, high-dimensional vector that condenses large amounts of patient information.

Although most of the CLaMs are freely accessible on the web, the best-performing models are trained on private datasets—EHR-BERT [34]; University of California, San Francisco–BERT [35]; and GatorTron [36]. Researchers developed the BERT for Biomedical Text Mining [37] and PubMedBERT [38] transformer models using biomedical literature from PubMed. NVIDIA developed the BioMegatron models in the biomedical domain with different sizes from 345 million to 1.2 billion parameters using a more expansive set of PubMed-derived free text [39]. Other large transformer models include ClinicalBERT [40], which has 110 million parameters and was trained using 0.5 billion words from the publicly available dataset.

## Structured Text Data

### Overview

In an effort to improve patient safety and reduce medical costs, >500,000 physicians and almost 6000 hospitals in the United States are expected to have operating EHRs and health IT systems [41] and charts [42] as of 2015. As a result, big data in health care in the United States have grown rapidly in recent years, with a massive database of electronic patient data. Among these, unstructured text data, also referred to as tabular data, are the most recorded and used time series, including numeric (eg, laboratory measurements and vital signs) and categorical (demographics, medications, and diagnostic codes) features. We note here that tabular data encompass a wide range of data, and we focus only on time series in this review.

### Preprocessing

Among time-series structured data, vital signs and laboratory results are the most common sources. These are typically represented in one of two ways: (1) as one 2D matrix in which each row is an encounter or patient and each feature can be represented across time as multiple columns, such as *heart_rate_time1* and *heart_rate_time2*; and (2) as stacked 2D matrices in which each matrix contains columns of all features at 1 time point. Depending on the recorded values, feature values can be either numerical or categorical. During preprocessing, the number of time points can be determined by the mean or median number of entries available across patients. The interval between each point can be absolute or relative time depending on whether the cohort has a large variability in terms of hospital stay time. With a large variability, a set number of time points can correspond to the relative time such that all patients have the same number of points but the intervals do not match up in absolute time. For uncommon measures such as certain laboratory tests, there may be large missingness within the cohort, which is further discussed in the next section. Finally, feature engineering can be performed through either a manual selection or feature selection algorithms [43,44].

### Applications

In this section, we introduce existing work on GenAI application in 3 main categories: data augmentation and imputation, disease classification and prediction, and counterfactual estimation.

#### Data Augmentation and Imputation

EHR datasets often face the problem of missing or unavailable data, which is especially common in laboratory data as not every patient in the cohort will receive the same tests. In addition, incomplete documentation or patient privacy concerns could also limit access to the complete dataset. Naive approaches such as filling missing data with 0 or forward filling in time add noise to the dataset at the cost of performance, prompting the need for better methods. Generative models impute missing values by learning underlying patterns of the trajectory, resulting in an overall smoother trajectory. Another challenge arises when the prevalence of certain rare diseases is so low that it is impossible for models to learn the pattern. In this case, generative models are used to generate synthetic patient records that mimic real-world data distributions. This can help in augmenting limited datasets and improving the robustness of predictive models trained on scarce data. The general approach of data imputation will start with an RNN model with LSTM layers [45-50] or GRU layers [51]. In more advanced approaches, researchers can modify the architecture parameters based on the dataset size or augment the model with additional pieces. In the work by Zaman and Du [52], the authors combined the GRU with ordinary differential equations to model irregularly sampled data and impute the entire time series. ImputeRNN [45] is a method that imputes data with consideration of medical bias by applying a mask during training. One can also combine DL methods, where a VAE is used first to find a data representation, which is then passed to an RNN for imputation [53]. For filling in missing data, it may be favorable to consider both previous and later observations. Bidirectional Recurrent Imputation for Time Series [54] imputes the missing data bidirectionally using an RNN graph. While RNN is the most common method in the space, GANs have also become a popular choice. In a few studies (4/155, 2.6%), time-series data were treated as an “image” on which a GAN was trained to fill in the missing “gaps” [55-58].

#### Disease Classification and Prediction

The most common goal in modeling disease trajectory is disease prediction and classification. Generative models can learn temporal dependencies within these sequences and generate realistic patient trajectories over time, enabling downstream predictive modeling of disease progression or treatment outcomes. There are several types of predictions that can be made: a binary classification of whether an event will occur (eg, mortality or sepsis), a probability prediction of an event occurring at some point, or a time at which an event occurs (survival analysis). Due to time-series data’s nature of unfixed length and frequency, many ways are proposed to handle such data. In earlier approaches, time points in time-series data are treated as separate entries with no relation to each other and passed through a fully connected neural network to predict the disease of interest. However, this approach ignores temporal information that can be crucial in future predictions. One of the most common ways of handling input with variable length is to first convert the input into a fixed-length representation, which is then passed through a network. In the study by Guo et al [59], measures such as BMI, smoking status, and cholesterol level were first converted to an embedding of fixed size and then passed through an RNN with LSTM layers to predict cardiac dysrhythmias. In another study [60], time-series data including laboratory results and vital signs were divided into 5 relative time points in reference to the duration of the hospital stay (eg, if the hospital stay for 1 patient is 4 hours, the times are admission, 1 hour into admission, 2 hours into admission, and so on) to fix the input length and predict mortality in patients with heart failure.

In many studies (19/155, 12.3%), once the embedding choice was made, the subsequent model of choice was similar. In these studies, RNN models were used to predict methicillin-resistant *Staphylococcus aureus* positivity [61], mortality [51,62-64], readmission [65-68], next diagnosis [69-72], length of stay [73,74], hypertension [75,76], treatment response [77], and survival analysis [78]. Furthermore, the general-use prediction models in several studies (4/155, 2.6%) [32,79-81] all used an RNN model to predict clinical events based on time-series data. Extending from a single prediction point, the work by Pham et al [81] used LSTM to model irregularly timed time-series data and predict multistep future trajectories on diabetes and mental health cohorts. In recent years, researchers have also turned to transformer models for prediction tasks [82-84] as they capture more long-range dependencies and provide more interpretability of model weights than RNNs. In the work by Sheikhalishahi et al [85], self-attention was used to measure each feature’s relevance to each other and also each time point’s relevance within each feature to predict delirium in critical care, and another study [86] used similar mechanisms to predict the mortality risk of patients with cardiac issues. Transformers have also been useful in making multi-point predictions. The Transformer for Electronic Health Records is one of the earlier models using transformers to predict clinical events and can predict up to 300 events simultaneously [87]. The Dual Event Time Transformer for Electronic Health Records is a general-purpose transformer-based method that takes in time-series features [88]. In addition to RNN and transformers, a deep diffusion model can also be used to learn complex representations of disease networks that can be used for downstream prediction tasks [89].

#### Counterfactual Estimation

Counterfactual estimations aim to track the “what-if” trajectory—what would a patient’s trajectories look like if they were not given any medication or treatment. To estimate a trajectory that is never observed, one must take into account both observed and hidden confounding variables, which is challenging in causal inference models. AI models offer the advantage of making no previous assumptions about the relationship between variables, learning purely from the data. However, this does not tackle the issue of the hidden variable, which is still an active problem in the field. Most commonly, RNNs were used to estimate treatment outcomes and, therefore, provide counterfactual estimation in several studies (4/155, 2.6%) [77,89-91]. Depending on the task and data at hand, modifications can be made to the basic RNN to achieve better results. In the work by Wang et al [92], a 2-layer bidirectional LSTM model was used for survival estimation. Similarly, the work by Li et al [93] combined RNN with g-computation, a causal inference method, to estimate treatment effects. A combination of reinforcement learning and RNN was also used to predict counterfactual evaluation in a study on public intervention for COVID-19 [94]. In addition to RNN models, autoencoders have been used to discriminate patient characteristics from treatment for counterfactual estimation [95].

### Model Selection

**Figure 5:**
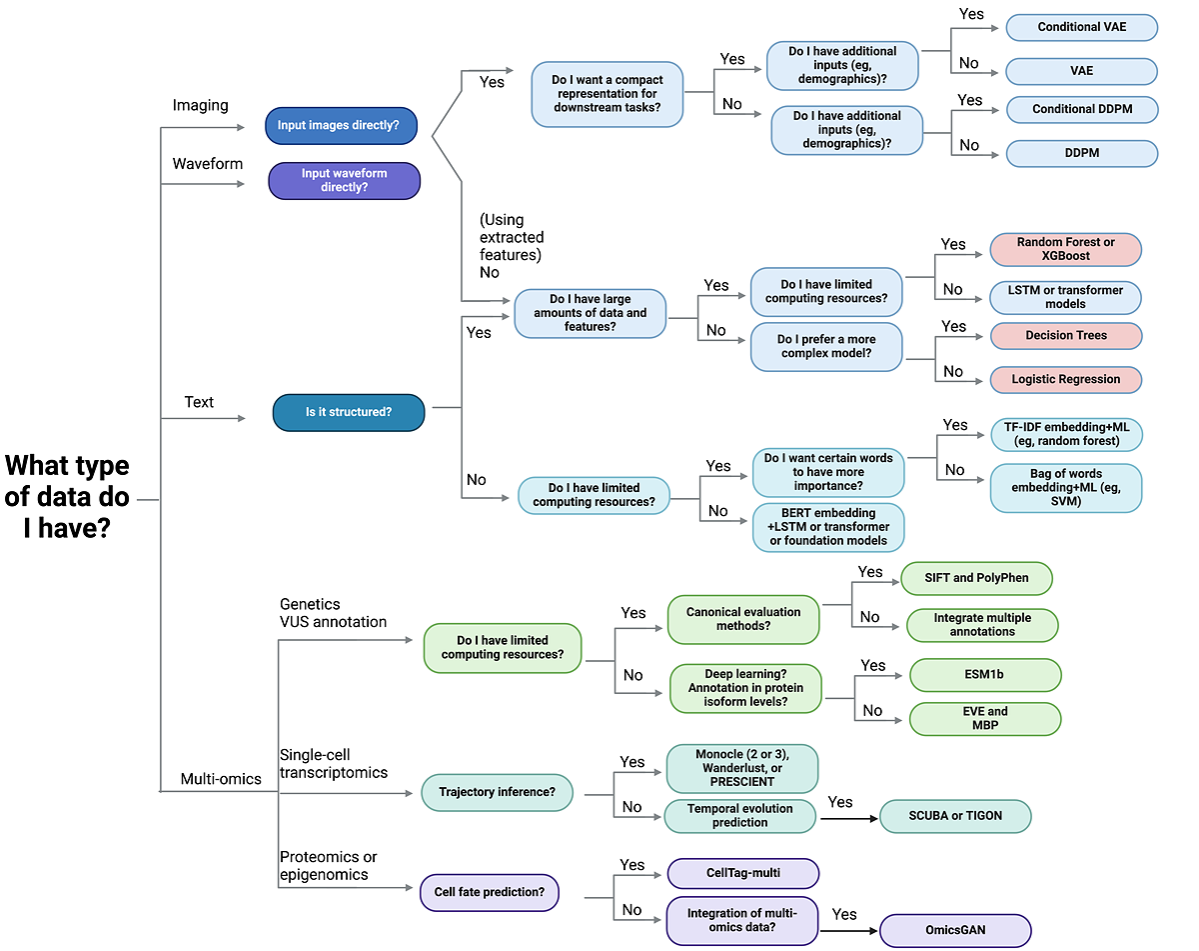
Flowchart to choose the appropriate deep learning model. BERT: Bidirectional Encoder Representations From Transformers; DDPM: denoising diffusion probabilistic model; ESM1b: Evolutionary Scale Modeling 1b; EVE: Evolutionary Model of Variant Effect; LSTM: long short-term memory; MBP: masked bidirectional prediction; ML: machine learning; PRESCIENT: Potential Energy Underlying Single-Cell Gradients; SCUBA: single-cell clustering using bifurcation analysis; SIFT: Sorting Intolerant From Tolerant; SVM: support vector machine; TF-IDF: term frequency–inverse document frequency; TIGON: Trajectory Inference With Growth via Optimal Transport and Neural Network; VAE: variational autoencoder; VUS: variant of uncertain significance; XGBoost: Extreme Gradient Boosting.

As they are most abundant and easiest to access, unstructured text data are a good place to start for clinicians who want to explore GenAI tools. In Figure 5, we present a flowchart to help guide the process of choosing the right model for the data and task at hand. The flowchart is organized in the same way as the sections in this paper, where different colors refer to different data modalities for easier visualization. In addition to the statistical methods mentioned previously, there are also a number of ML models, including logistic regression, decision tree, random forest, and Extreme Gradient Boosting (colored red in Figure 5). While we do not discuss these methods in this review, there are review papers that describe them in detail [96-98]. If the dataset is quite small, a traditional autoregressive or a simple ML method would work better as more complex models require more data. However, if the dataset is large, DL models would perform better as the relationship between features is more complex and tends to be nonlinear. Commonly, the model of choice will be RNN or transformer based, which also have the most literature available. Once one has chosen a base model, they can start with a published framework and modify certain parameters based on their goal. If the model is quite simple and only consists of linear layers, one may also consider changing part of the structure to newer architectures, such as attention layers, or chaining 2 models together to improve performance.

### Challenges and Future Directions

As ML and DL methods, including GenAI, continue to grow with an increasing amount of available data, many comparisons have been made between AI methods and traditional statistical methods. Cerqueira et al [99] compared ML methods in time-series forecasting (rule based, random forest, and Gaussian process regression) to statistical methods (ARIMA, Naive2, and ETS) using datasets with varying sample sizes up to 1000 and showed that ML methods outperformed statistical methods when the sample size was relatively large. Makridakis et al [100] jointly evaluated the accuracy of DL methods and statistical methods (Naive2, ARIMA, ETS, and ensemble methods) using the 1045 M3 dataset and showed that, among the nonensemble approaches, the statistical methods on average yielded better performance than DL methods for short-range prediction with regard to symmetric mean absolute percentage error and mean absolute scaled error, whereas the DL methods performed better in general for medium- and long-range predictions. The DL ensemble method using a combination of the 4 DL methods consistently outperformed the nonensemble methods in the study by Bonnheim et al [101]. However, in a practical setting, AI methods face the biggest challenge of low transparency due to their black-box nature. Interpretability has been an active area of research, and many techniques and tools have been proposed to understand the decision-making process of such models. Feature importance analysis tools such as Shapley Additive Explanations [102] and Local Interpretable Model-Agnostic Estimations [103] estimate feature contribution to reveal top features that are important to the decision. These tools may help shed light on how the model predicts the outcome, which is extremely important in the field of health care. A second challenge that AI methods face is the trade-off between generalization and performance as an exceptionally good performance of a model on one dataset may be caused by overfitting. As patient distributions can differ widely between hospitals and areas, the model’s robustness must be measured before deploying it in practice. Nonetheless, GenAI has shown great potential in clinical support, such as early warning systems or care assistance tools. Future GenAI tools that tackle these challenges will become paramount in health care.

## Unstructured Text Data

### Overview

Unstructured data include a wide range of clinical narratives, such as progress notes, radiology reports, patient correspondence, and discharge summaries, comprising 80% of EHRs [104]. The loosely structured nature of typed text (also known as “free text”) is effective in day-to-day clinical workflows but presents a major challenge for automating EHR-based registries. The unstructured data may contain key patient information missing in structured data, extra information complementing structured data, or even data that may contradict information presented in structured data. As such, the overall goal of modeling using unstructured data is to assist disease prediction using structured data by adding critical missing information. The complexities of unstructured data, along with the fact that existing text-mining tools and NLP applications have limited accuracy in extracting information from free text, have prompted some registries to ask for a manual chart review of individual patients before final inclusion in the registry. Unstructured data limit the application of automated computational phenotyping methods and increase the likelihood of low data quality (eg, missing data) when data are extracted from structured EHR data only [105,106].

### Preprocessing

Information retrieval from unstructured data is challenging because the information can often be of low quality and contain missing values. Commonly used data preprocessing techniques for unstructured data include data collection, data cleaning, tokenization, stemming, and the removal of stop words [107,108]. Tokenization refers to segregating, for example, words and punctuation marks in the input document as tokens and computing their frequency. Stemming is the process of identifying the roots of words, typically by removing the suffix. Stop word removal consists of removing infrequent words to reduce the dimensionality of the dataset. Once the key components of the dataset are extracted, they can be used for downstream applications such as the identification of risk factors and individuals at high risk of a particular disease.

### Applications

As the application of large language models to health care time-series data is still a developing area of research, in this section, we discuss static time applications of these models. We will discuss potential research directions for time-series unstructured text based on progress in other fields such as climate and finance in the Challenges and Future Directions section. Both traditional NLP and FMs can be used to perform clinical NLP tasks such as clinical concept extraction (or named entity recognition), medical relation extraction, semantic textual similarity, natural language inference, medical question answering, and medical report summarization. The domain of medical report summarization is a popular area of research, with numerous papers published on the use of NLP and FMs to summarize medical reports, health care records, and medical dialogues [109]. A radiology report is a medical document that contains the details of an imaging examination (such as x-rays and magnetic resonance imaging [MRI]). A radiology report consists of three components: (1) the Background section, which contains the medical history of the patient; (2) the Findings section, which discusses the crucial observations and findings of the radiology examination; and (3) the Impression section, which is a short summary of the findings section. The Impression section is usually written by medical professionals, which is a time-consuming process. The only aim of radiology report summarization is to automate the generation of this Impression section. Several attention-based and FM-based models have been developed to improve the quality of summarization [110].

Medical dialogue summarization corresponds to the automatic generation of coherent summaries that capture medically relevant context from dialogues between patients and health care providers. Medical dialogue summarization can help medical providers keep a record of patient encounters and also provide the necessary context of a patient’s medical history during patient handoffs between providers. Existing studies have used techniques from computational linguistics [111], NLP (Pegasus [112]), pretrained language models, and low-shot learning to collect labeled data and perform medical dialogue summarization.

Medical question answering refers to developing a technique that automatically analyzes thousands of articles to generate a short text, ideally in less than a few seconds, to answer questions posed by physicians [113]. Such a technique provides a practical alternative that allows physicians to efficiently seek information at the point of patient care. Several question-and-answer systems have been developed using NLP [114] and FMs (generalist medical AI and MedLM).

### Model Selection

FMs hold great potential to assist clinicians in a wide range of health care problems. However, clinicians should be aware of the risks associated with the use of these models and potential data leaks. They should also select which models to use based on the computational resources available to them, as shown in Figure 5, as the training of these models is becoming increasingly expensive (Table 1).

### Challenges and Future Directions

Compared to traditional ML models, FMs have a significantly higher computational cost, requiring massive datasets and graphics processing unit specifications for training. While they achieve high predictive accuracy [115], these models are composed of millions to billions of parameters and have poor interpretability and high latency and runtime. While these costs can be lowered over multiple downstream applications, their value may take longer to realize than that a smaller model developed for a single high-value task [30]. In addition, data privacy and security are significant concerns with FMs as they may leak protected health information through model weights or prompt injection attacks [116]. As the cost of computing decreases and the amount of data increases, FMs may become easier to train and more accessible. One active area of research is testing FMs’ potential in multimodality input, in which FMs are trained and evaluated on multimodal datasets, such as both text and images. These models can be used to generate personalized treatment plans based on the patient history as well as develop early warning systems for individuals at high risk. These advancements would transform how health care professionals interact with patient data, making health care more predictive, personalized, and precise.

## Medical Imaging

### Overview

Medical imaging refers to 2D or 3D images obtained from radiology procedures such as MRI and computed tomography (CT). GenAI has been applied to a wide set of problems in medical imaging to solve issues of scarcity, heterogeneity between datasets, and low resolution quality, among others. There are 3 main classes of generative vision methods: GANs, VAEs, and DDPMs. Each of these classes has been applied to medical imaging. Among all applications, medical image series input has been used mostly in data generation, anomaly detection and classification, and image segmentation and registration tasks.

### Preprocessing

There are several steps in preparing a medical imaging dataset as the quality varies largely depending on location, time, and cohort [117]. First, a denoising technique is applied to remove as much nuisance noise as possible. After denoising, data interpolation can be conducted for certain images such as CT scans to ensure the same spacing across all images. In the case of MRI images, bias field correction may be performed to adjust image intensity. Third, image registration is performed to align all images with a common template to avoid unnecessary incoherency among images, such as rotation. Finally, images can be standardized or normalized to improve algorithm efficiency. Depending on computational resources, the image may also be downsampled to a lower resolution or split into patches to reduce computational cost. Publicly available datasets such as those of the Alzheimer’s Disease Neuroimaging Initiative [118] and Open Access Series of Imaging Studies [119] do not need much preprocessing as they have been carefully curated. However, image registration is recommended to align all patients with a common template of choice if multiple datasets are combined. The medical images are usually represented as 2D matrices or 3D vectors of the pixels or voxels.

### Applications

#### Data Generation

Similarly to tabular data, data generation has been used to combat the problem of data scarcity. For longitudinal medical image series, GANs, VAEs, and DDPMs have all been used to generate synthetic images. In the work by Hamghalam and Simpson [120], a recurrent conditional GAN was used to generate sequential image data conditioned on past variables. Diffusion-based models have also been applied to impute longitudinal CT and MRI images [121]. Finally, predicting and generating aging brain images using only the input of age and disease state is an example of applying autoencoders with contractions on the latent space [122].

#### Anomaly Detection and Classification

The most common application to longitudinal images is anomaly detection and disease progression prediction. For clinical diagnosis, GenAI-based anomaly detection tools have been developed in which the model identifies deviations in 1 image from the rest of the population, aiding in the detection of diseases or abnormalities. For medical image series, previous scans are used as inputs or conditional variables to predict the next image. Many approaches in this space are a variation of GANs as they were introduced earlier than VAEs and DDPMs. Gaussian-Poisson GAN uses stacked GANs to predict tumor growth from longitudinal MRI [123]. The multi-Pareto GAN makes longitudinal prediction of infant MRI using multi-contrast perceptual adversarial learning [124]. In the low-dimensional GAN [125], disease progression images are predicted using missing MRI in the input. In the study by Devika et al [126], the authors trained a GAN to predict autism spectrum disorder given longitudinal structural MRI scans. More recently, VAEs have been applied extensively as they offer a latent space to work with. In general, researchers start with a vanilla VAE and apply slight modifications to fit their data modality and goal to detect and predict diseases, including multiple sclerosis [127], Alzheimer disease (AD) [128-131], tau biomarker detection for AD [132], glaucoma [133], and lung cancer [134]. There has also been an increase in works published using multi-modality data, such as imaging data and tabular or genetics data. In the work by Kmetzsch et al [135], the authors trained a VAE model to predict disease progression score based on longitudinal images and micro-RNA data input. When dealing with multimodal inputs, a combination of DL models dealing with each modality may also be advantageous. The work by Sauty and Durrleman [136] combined VAE with a linear mixed-effects model to learn a Riemann progression model that can be applied to general disease trajectory estimation. Another study combined an autoencoder framework with attention units in a transformer to predict final ischemic stroke lesions from MRI [137]. In the study by Zhang et al [138], a spatiotemporal convolutional LSTM was learned to combine imaging and nonimaging data to predict tumor growth images. Conditional SliceGen is a method that takes an arbitrary axial slice in the abdominal region as a condition and estimates a vertebral-level slice [139].

#### Image Segmentation and Registration Tasks

Another common task in medical imaging is image segmentation and registration. We note here that, while image segmentation and registration tasks are different concepts, the underlying models applied to solve the problems are largely the same. In the work by Zhang et al [140], an encoder-decoder structure model was used for contrastive learning for image segmentation. Extending from autoencoders, a conditional VAE has also been used to produce cardiac image segmentation conditioned on predefined anatomical criteria [141]. We note here that, while work in this area using time-series input is scarce, the underlying model and training process remain the same for single time input models [142-147]. Furthermore, many of the methods mentioned previously for anomaly detection can also be applied to registration and segmentation tasks during training.

### Model Selection

Although there are many tasks associated with imaging data input, the underlying model architecture remains largely the same for prediction, synthesis, registration, or even domain transfer tasks. For anyone using time-series or longitudinal images for any task, choosing 1 of the 3 families of vision methods following the workflow in Figure 5 is a good place to start. One may also refer to Table 1 to compare the models’ strengths and weaknesses. GANs are an earlier framework that remains powerful today, especially with synthetic generation, but are more complicated to implement as they have a generator and a discriminator. On the other hand, VAEs offer a latent space that can be used to downstream linear models such as mixed-effects models to control for variables such as age, but its generation quality may not be as ideal as that of GANs or DDPMs. Finally, diffusion models have been largely applied in recent years due to their simple architecture compared to that of the other 2 and low memory requirements but offer very little interpretability on the generation process. For clinicians with limited computing resources, 3D images may be too large to fit onto graphics processing units, and in many cases, the images can be split into smaller patches to reduce memory footprint [148]. Finally, there are publicly available general-purpose vision models that can be downloaded as a starting point, which can be further trained and fine-tuned for the specific task at hand.

### Challenges and Future Directions

With computer vision models’ success in recent years, there is a lot of potential in their applications to medical imaging. In recent years, diffusion models have shown great promise in generating images that are comparable to those generated by state-of-the-art GAN methods. Furthermore, GenAI methods that support video generation can potentially be adapted for medical imaging [149-153]. However, some issues that are present in current vision models also extend to medical imaging, such as illogical generations that do not comply with physical laws [119]. Therefore, clinicians should take the generations with a grain of salt when applying them to downstream tasks. The advent of vision models has been revolutionary in the study of medical images as previous methods must first transform the images into numeric representations, such as thickness and mean intensity. These representations are then fed into linear models for tasks such as classification and segmentation. To work with raw image inputs, linear models of estimation such as principal component analysis or single-value decomposition are often used. However, these generations often have poor resolution or fail to adequately capture variability. In contrast, modern neural network approaches (eg, CNN) *jointly* learn the optimal feature extraction strategy together with the downstream task. Thus, GenAI algorithms are able to consider highly nonlinear and nuanced interactions between inputs that better capture the underlying distribution of the data. While GenAI faces the challenge of transparency, interpretability methods such as Gradient-Weighted Class Activation Mapping [154] have shown convincing results. Another challenge of GenAI in medical imaging is the computational resources required to support such a model, which will become cheaper and easier to acquire as hardware technology becomes more efficient.

## Physiological Waveforms

### Overview

Waveform data capture the change of one or multiple physical quantities over time and can be viewed as either univariate or multivariate time-series data. Examples of medical waveform data include single-lead electrocardiograms (ECGs) or photoplethysmograms, which can be represented as univariate time series. Multi-channel technologies such as electroencephalograms (EEGs) are typically represented as multivariate time series. Although waveforms are considered to be closer to structured time-series tabular data, DL methods applied to structured data often face technical limitations when applied to waveforms as the sampling frequency is much higher. As such, GenAI methods applied to waveforms often resemble speech processing [155] or vision-based models adapted for 1D input signals.

### Preprocessing

Physiological waveforms are often recorded over a short period with low missingness but face the problem of noise and signal artifacts. Therefore, waveform preprocessing aims to remove as much nonphysiological noise and artifacts as possible while preserving the actual signal. This is typically accomplished by applying digital filters designed to address nonbiological signals, drifts, and spikes [155]. Depending on the signal, specific filters have also been developed and shown strong performance, such as the Wiener filter for ECGs [156]. After filtering, the waveform is usually normalized for efficiency and resampled to a common frequency.

### Applications

GenAI models have gained increasing popularity among medical waveform data. Major tasks using GenAI models for this type of data include synthesis and imputation and anomaly detection.

#### Synthesis and Imputation

GenAI-based approaches can augment existing data and generate synthetic data to support downstream algorithms, allowing for more applications when the amount of real medical waveform data is limited. For this task, GANs have been used extensively [156-161], along with diffusion models [162]. In their work, Elazab et al [123] made modifications to the improved Wasserstein GANs and generated single-channel EEG signals. Zhu et al [163] constructed a GAN with bidirectional LSTM and CNN to synthesize single-beat ECGs and demonstrated that the model performed consistently better than benchmark architectures with regard to the quality of the signals. In another work, To et al [127] reconstructed high–sampling sensitivity EEG signals with improvements in reconstruction and classification results.

#### Anomaly Detection

While little work has been done on anomaly detection directly, GAN-based approaches have been explored to detect cardiac abnormalities [164-166] and seizures [167,168]. Diffusion-based models have also been used for anomaly detection with ECGs and EEGs. Xiao et al [169] adopted a denoising diffusion-based imputation method with conditional weight-incremental diffusion to improve data generation quality and ECG anomaly detection stability. Wang et al [170] designed a diffusion-based framework with a reverse diffusion data augmentation module to learn more generalized features for major depressive disorder detection using EEGs.

### Model Selection

In general, waveform data are best trained using vision models such as VAE and DDPM as the time series can be viewed as a 1D image. Therefore, the workflow to choose an appropriate model for waveform data is similar to that for imaging data. As GANs are often more complex but do not significantly outperform other vision models, they are being replaced with VAEs and DDPMs [171,172].

### Challenges and Future Directions

As GenAI models are only beginning to be applied to waveform data, there are many directions to pursue. One of these is cross-modality imputation, where, given an EEG, a GenAI model can be trained to generate the corresponding functional MRI or vice versa. Another area of study is to convert EEG signals to natural language by combining vision models with a language model [173]. As previous studies have focused on modeling a single type of waveform data in isolation, one natural direction is to use more data sources, for example, EEGs with text data or ECGs with medication data, as they are often informative about one another in tasks such as imputation [174] and forecasting [175].

## Genetics and Multi-Omics Data

Multi-omics data encompass genomics, transcriptomics, proteomics, epigenomics, and metabolomics, offering a detailed picture of biological activities at various molecular layers. One of the most successful applications of AI in genetics is in trajectory modeling using multi-omics data, which focuses on how biological systems such as cell development, diseases, or reactions to treatments change over time.

### Preprocessing

Multi-omics data comprise multiple-sequencing data types and various features. To efficiently preprocess the data, different methods are used for each type of data. DNA sequences are represented as strings of adenine, thymine, guanine, and cytosine nucleotides, which can be transformed into a numerical matrix in which each nucleotide is represented as a binary vector. For transcriptomics, expression data are often represented as a matrix of continuous numeric values in which each row corresponds to a sample or a cell and each column corresponds to a gene or transcript. Proteomics data are similarly structured but represent peptide abundances or protein expression levels, and epigenomics data such as methylation status are typically captured as binary values or continuous levels indicating methylation at specific sites. Depending on the goal, different omics data can be combined to uncover time-related trends and critical factors that influence those biological shifts.

### Applications

#### Identifying Pathogenic Mutations

Identifying pathogenic mutations is pivotal for unraveling the genetic underpinnings of diseases. The task of variant effect prediction, which seeks to determine the phenotypic implications of genetic variations, stands as a cornerstone challenge in human genetics. Traditional approaches leveraging statistical methods alongside evolutionary conservation scores such as Sorting Intolerant From Tolerant and phyloP have made strides in classifying variants of uncertain significance (VUSs) [176,177]. Despite these advancements, accurately differentiating between variants that disrupt protein function and those with neutral effects remains a daunting challenge. The persistence of many missense variants within the gray area of VUSs significantly hampers the diagnostic utility of exome sequencing in clinical settings. To effectively address the challenge of categorizing mutations, particularly those classified as VUSs, ML techniques have been increasingly applied. Among the array of ML methodologies, random forest, gradient-boosting models, and support vector machines are often used for training complex genetic datasets to decipher VUSs [178,179]. However, the high dimensionality of genomic information and the intricate interactions between genetic variants often exceed the capacity of traditional ML techniques, leading to potential overfitting and a lack of generalizability to unseen data. DL methods, including CNNs and RNNs, offer a promising solution for addressing the challenges of modeling complex, nonlinear relationships in high-dimensional biological data. These methods significantly diminish the need for manual feature engineering by efficiently learning from raw data. Among the DL models developed for predicting VUSs are Evolutionary Scale Modeling 1b (ESM1b), Evolutionary Model of Variant Effect, MetaRNN, masked bidirectional prediction, PrimateAI, and deleterious annotation of genetic variants using neural networks. ESM1b is particularly noteworthy for its innovative use of a protein language model developed by Meta AI [180-185]. ESM1b has demonstrated exceptional capability in classifying approximately 150,000 ClinVar and Human Gene Mutation Database variants and annotating approximately 2 million variants as damaging in specific protein isoforms, a feat not previously achieved with ML-based methods. This focus on isoform-specific annotations underlines the critical importance of considering all protein isoforms in variant effect predictions, marking a significant advancement with which ESM1b contributes to genetic research.

#### Trajectory Modeling

Time-series transcriptomics and proteomics data have been used widely to study trajectories in biological processes and disease development. Among them, single-cell transcriptomics data have been used to reveal cellular dynamics via pseudotime inference models, including Monocle (versions 2 and 3), Wanderlust, scVelo, single-cell clustering using bifurcation analysis, partition-based graph abstraction, and Trajectory Inference With Growth via Optimal Transport and Neural Network [154]. Monocle uses single-cell variations to sequence cells in pseudotime, depicting their progression through biological processes such as differentiation based on gene expression. Monocle version 2 [186] uses reversed graph embedding with a minimum spanning tree algorithm for pseudotime reconstruction, whereas Monocle version 3 [187] enhances this with principal graph learning for refined trajectory inference. Wanderlust adjusts each cell’s trajectory position using weighted averages from the shortest-path distances of randomly chosen waypoints until convergence, thereby producing an average trajectory. scVelo [188], a likelihood-based dynamic model, estimates RNA velocity to derive dynamic insights from RNA sequencing data, analyzing gene-level transcriptional dynamics by determining gene-specific transcription, splicing, and degradation rates, which is suitable for both transient states and systems with varying subpopulation kinetics. Single-cell clustering using bifurcation analysis [189] forecasts the temporal evolution of gene expression in single cells using theories of nonlinear dynamics and stochastic differential equations, aiding in the comprehension of gene expression dynamics. Partition-based graph abstraction [190] merges clustering and trajectory inference in single-cell RNA sequencing data, creating a graph to depict cell relationships based on gene expression and enhance understanding of cellular transitions. Finally, Trajectory Inference With Growth via Optimal Transport and Neural Network [191] uses dynamic unbalanced optimal transport based on the Wasserstein-Fisher-Rao distance to integrate temporal datasets, which provides a framework for connecting temporal measurements and predicting novel dynamics. In addition, expression data can be used to study responses to infectious diseases. Huang et al [192] analyzed time-series expression data from human volunteers infected with influenza. They identified distinct temporal patterns of gene expression that could discriminate between asymptomatic and symptomatic infections. Similar approaches have been used to study the response to various treatments, such as interferon-β therapy in patients with multiple sclerosis [193].

On the other hand, single-cell proteomics approaches such as mass cytometry (cytometry by time of flight) have revolutionized our ability to understand complex cellular processes at an unprecedented resolution. Palii et al [194] used single-cell proteomics to define the temporal hierarchy of human erythropoiesis. The analysis revealed a timely ordered appearance and disappearance of transient cell populations or stages that accumulate at various positions along the erythroid trajectory, with cells undergoing gradual transitions between these stages. In the fields of proteomics, epigenomics, and metabolomics, CellTag-multi [195], a method for single-cell lineage tracing across single-cell RNA sequencing and single-cell assay for transposase-accessible chromatin sequencing assays, mapped transcriptional and epigenomic states of progenitor cells, significantly improving cell fate prediction. In addition, linear mixed models have been used to quantify temporal changes in metabolite concentrations, offering insights into the dynamics of the metabolic system [196,197].

### Model Selection

GenAI has shown great promise in analyzing multi-omics data for disease progression analysis. By using techniques such as omicsGAN, which uses GANs, and graph-linked unified embedding and MultiVI, which use VAEs, GenAI facilitates the integration and compression of multi-omics data from diverse sources into a unified representation [198-200]. This aids in a more comprehensive analysis of the data. In addition, tools such as Potential Energy Underlying Single-Cell Gradients apply generative modeling to map out potential landscapes from time-series single-cell transcriptomics data, making it possible to generate trajectories for unseen data points [201]. This opens up avenues for hypothesizing about biological perturbations and pathways of disease progression. Moreover, GenAI proves instrumental in identifying distinct disease subtypes and stratifying patients based on their multi-omics profiles. For example, gene-guided weakly supervised clustering via GANs uses a GAN to generate imaging features from brain MRIs and single nucleotide polymorphisms to explore conditions such as AD and hypertension [202], whereas the Trained GAN Discriminator model stratifies patients with breast cancer into high- versus low-risk categories based on their transcriptome profiles [203]. For clinicians interested in working with multi-omics data, we outline a procedure for finding the best tool in Figure 5 based on their specific needs, such as resource availability and the type of data analysis required, including genetics, single-cell transcriptomics, and proteomics or epigenomics.

### Challenges and Future Directions

Analyzing and modeling multi-omics data in time-series contexts presents challenges in addressing missing data across different biomolecule types, which complicates the integration of datasets obtained through various omics technologies. In addition, the complexity of biological interactions can lead to analytical difficulties when dealing with high-dimensional data. The integration of multi-omics data is becoming increasingly important in the pursuit of future progress. This approach uses various biological data layers—genomics, transcriptomics, proteomics, and metabolomics—to enhance the understanding of genetic interactions and disease mechanisms.

## Discussion

### Data Ownership and Privacy

#### Overview

While GenAI software has proven powerful in understanding and predicting disease trajectories across modalities, its implementation in the clinic remains difficult due to challenges including ethical considerations [204] and other regulatory and technical challenges. Regulations such as HIPAA (Health Insurance Portability and Accountability Act) and the General Data Protection Regulation protect personal health information, but the evolving nature of GenAI complicates compliance, especially regarding AI-generated synthetic data and indirect identification risks [205-207]. This rapid advancement outpaces regulatory updates, causing uncertainty among health care providers and AI developers about legal responsibilities and best practices [208-210]. Technically, health care time-series forecasting struggles with short data lengths and significant temporal changes as discussed in previous sections, making model training and parameter estimation difficult [211,212].

Data ownership and privacy in health care GenAI are critical issues involving rights and responsibilities over data control, use, and dissemination [213]. This includes patient records, genetic information, and AI-generated datasets. Ambiguities in ownership, particularly with AI-generated synthetic data, complicate matters. This raises questions about who owns the data—the AI developer, health care providers, or patients [214]. Current regulations enforce data minimization, purpose limitation, and consent to protect patients’ privacy rights [215]. However, GenAI models require extensive patient data for training, leading to the risk of reidentification of anonymized data through advanced algorithms [216]. Ensuring that raw patient data remain protected is crucial to prevent unauthorized access [217,218].

#### Security

AI models in health care should be deployed with the highest standard of data security as breaches of sensitive patient information can be disastrous. In 2023, >500 data breaches affected >112 million individuals [219]. Cyberattacks such as phishing and malware are common, but prompt injection attacks in GenAI models are emerging threats [220]. Effective countermeasures such as prompt filtering and human-in-the-loop systems are essential despite potentially compromising model performance [221-224].

#### Bias

The deployment of GenAI in health care can also result in inadvertently embedded societal biases, leading to discriminatory outcomes [225]. Biases in training data can result in disparities in treatment across different patient groups [226]. Addressing these biases requires comprehensive strategies throughout AI development to ensure fairness and equity [227-231].

#### Clinical Safety and Reliability

Ensuring clinical safety and reliability is vital for the trustworthiness of health care GenAI. AI models can produce inconsistent or erroneous outputs, particularly in complex medical scenarios, due to issues such as hallucinations [232,233]. In health care, such errors are unacceptable and can lead to harmful consequences, which require rigorous scrutiny and validation. Moreover, AI models, particularly DL models, often lack transparency and explainability, posing challenges in health care [234]. Enhancing transparency and explainability through techniques such as feature importance scores and decision trees is crucial for gaining trust and ensuring comprehensible AI decisions [235-237]. Regulatory and ethical frameworks must evolve to support these efforts [238].

### Conclusions

While there remain many challenges to deploy reliable GenAI methods in real practice, including technical, regulatory, and ethical issues, these models can have huge impacts that could benefit patients, practitioners, and administrators. As such, collaboration among policy makers, legal experts, AI developers, and health care providers is crucial in overcoming these issues. In this paper, we introduced concepts basic to DL and GenAI to those unfamiliar with the field, reviewed existing work on GenAI application to time-series health data, and offered suggestions to clinicians who are interested in applying such tools to their field. We hope that this review bridges the gap and promotes better collaboration between technical and applied disciplines to develop better methods that are suitable and robust in a practical setting.

## Appendix

Multimedia Appendix 1 PRISMA-ScR (Preferred Reporting Items for Systematic Reviews and Meta-Analyses extension for Scoping Reviews) checklist.

Multimedia Appendix 2 Summary of existing reviews on artificial intelligence in health care.

Multimedia Appendix 3 Number of existing reviews and surveys published after 2010 identified from the preliminary search by year (before filtering) using the following artificial intelligence– and biomedical-related search string: (“Generative AI” OR “Generative artificial intelligence” OR “genAI” OR “foundation model” OR “GPT” OR “Generative Adversarial Networks” OR “transformer” OR “variational autoencoder” OR “diffusion model” OR “flow model”) AND (“review” OR “summary” OR “survey”) AND (“time series”) AND (“disease trajectory” OR “health care”).

## Abbreviations

AD: Alzheimer disease
AI: artificial intelligence
ARIMA: autoregressive integrated moving average
BERT: Bidirectional Encoder Representations From Transformers
CLaM: clinical language model
CNN: convolutional neural network
CT: computed tomography
DDPM: denoising diffusion probabilistic model
DL: deep learning
ECG: electrocardiogram
EEG: electroencephalogram
EHR: electronic health record
ESM1b: Evolutionary Scale Modeling 1b
ETS: exponential smoothing
FEMR: foundation model for electronic medical records
FM: foundation model
GAN: generative adversarial network
GRU: gated recurrent unit
HIPAA: Health Insurance Portability and Accountability Act
LSTM: long short-term memory
ML: machine learning
MRI: magnetic resonance imaging
NLP: natural language processing
PRISMA-ScR: Preferred Reporting Items for Systematic Reviews and Meta-Analyses extension for Scoping Reviews
RNN: recurrent neural network
VAE: variational autoencoder
VUS: variant of uncertain significance
