# Supplementary figures and images for: Generative AI Models in Time-Varying Biomedical Data: Scoping Review
Source: J Med Internet Res. 2025 Mar 10;27:e59792. doi: 10.2196/59792 (PMC11933772; doi:10.2196/59792)

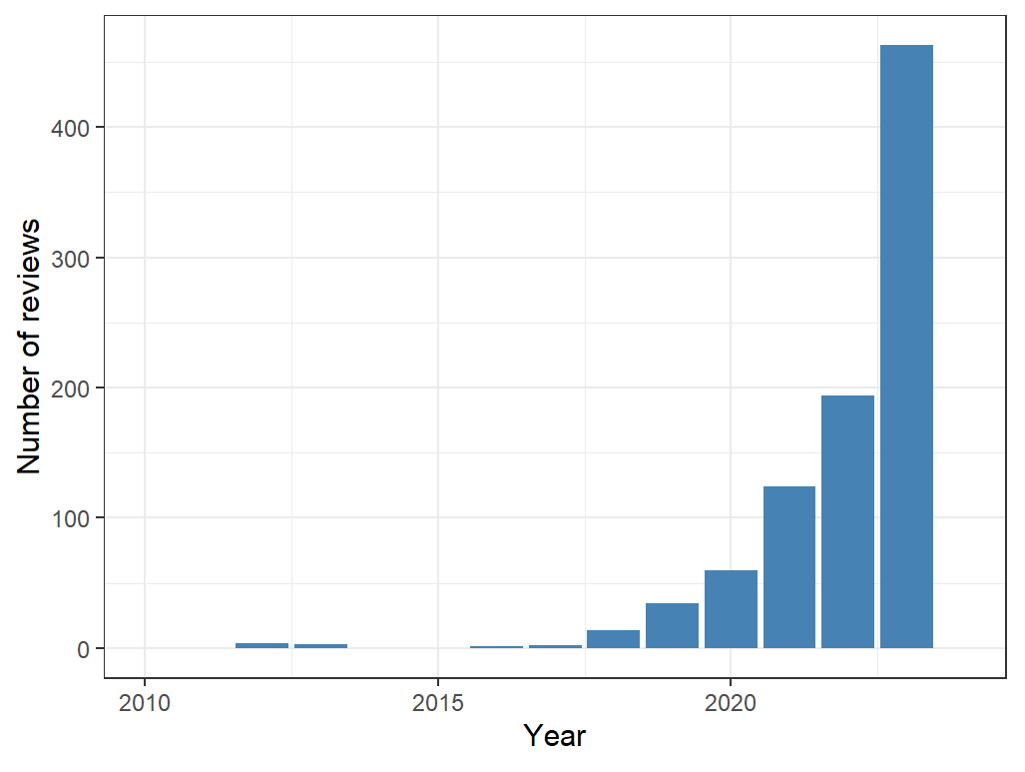

Supplement: Multimedia Appendix 3 [file jmir_v27i1e59792_app3.png]
